# Supplementary material for: Cword2vec: a novel morphological rule-based word embedding approach for Urdu text sentiment analysis
Source: PeerJ Comput Sci. 2025 Jul 15;11:e2937. doi: 10.7717/peerj-cs.2937 (PMC12453651; doi:10.7717/peerj-cs.2937)
Supplement: Supplemental Information 3 [file peerj-cs-11-2937-s003.docx]

| Compound Type | Construction | Example |
| --- | --- | --- |
| Noun (N) | N + ADJ | آب شیریں (sweet water) |
|  | N + N | رام چندر (Ram Chandar) |
|  | N + Prep + N | ریل کا انجن (train engine) |
|  | N + vowels + N | زمین و آسمان (earth and sky) |
|  | N + N | چالیس سپاہی (forty soldiers) |
| Verb (V) | Verb + Verb | یقین کرنا (to believe) |
| Adjective (ADJ) | ADJ + N | مرِد دانا (clever man), |
|  | ADJ + Prep + N | تیز دھوپ میں گرمی  (Heat in the hot sun) |
|  | ADJ + Verb | تیز دوڑنا (run fast) |
|  | ADJ + ADJ | عرق گلاب (rose water) |
|  | Adverb + consonant adverb | آتش کدہ (hearth) |
| Preposition | Preposition + postposition | لاجواب (fantastic) |
| Mohmil Compounds | Meaningful word + meaningless word | کھانا وانا (eating) |
| Hybrid Compounds | First Urdu and second English word | کریانہ سٹور (grocery store) |
| Partial Reduplication | Word + word with missing first character | گائے بگائے (sometome) |
| Reduplication Compounds | Word + Word | قدم قدم (step by step) |
| Reduplication Compounds | Word + Word | آب شیریں (sweet water) |
