# Supplementary material for: Cword2vec: a novel morphological rule-based word embedding approach for Urdu text sentiment analysis
Source: PeerJ Comput Sci. 2025 Jul 15;11:e2937. doi: 10.7717/peerj-cs.2937 (PMC12453651; doi:10.7717/peerj-cs.2937)
Supplement: Supplemental Information 4 [file peerj-cs-11-2937-s004.docx]

| Source Text | Training Samples |
| --- | --- |
| **شہری زندگی** مکمل طور پر مفلوج ہو کر رہ گئی۔ | (مکمل طور, شہری زندگی) |
| **شہری زندگی** مکمل طور پر مفلوج ہو کر رہ گئی۔ | (مکمل طور پر مفلوج, شہری زندگی) |
| شہری زندگی **مکمل طور** پر مفلوج ہو کر رہ گئی۔ | (مکمل طور پر مفلوج, مکمل طور) |
| شہری زندگی **مکمل طور** پر مفلوج ہو کر رہ گئی۔ | (شہری زندگی, مکمل طور) |
| شہری زندگی **مکمل طور پر مفلوج** ہو کر رہ گئی۔ | (مکمل طور پر مفلوج، مکمل طور) |
| شہری زندگی **مکمل طور پر مفلوج** ہو کر رہ گئی۔ | (مکمل طور پر مفلوج، شہری زندگی) |
