# Supplementary figures and images for: Cword2vec: a novel morphological rule-based word embedding approach for Urdu text sentiment analysis
Source: PeerJ Comput Sci. 2025 Jul 15;11:e2937. doi: 10.7717/peerj-cs.2937 (PMC12453651; doi:10.7717/peerj-cs.2937)

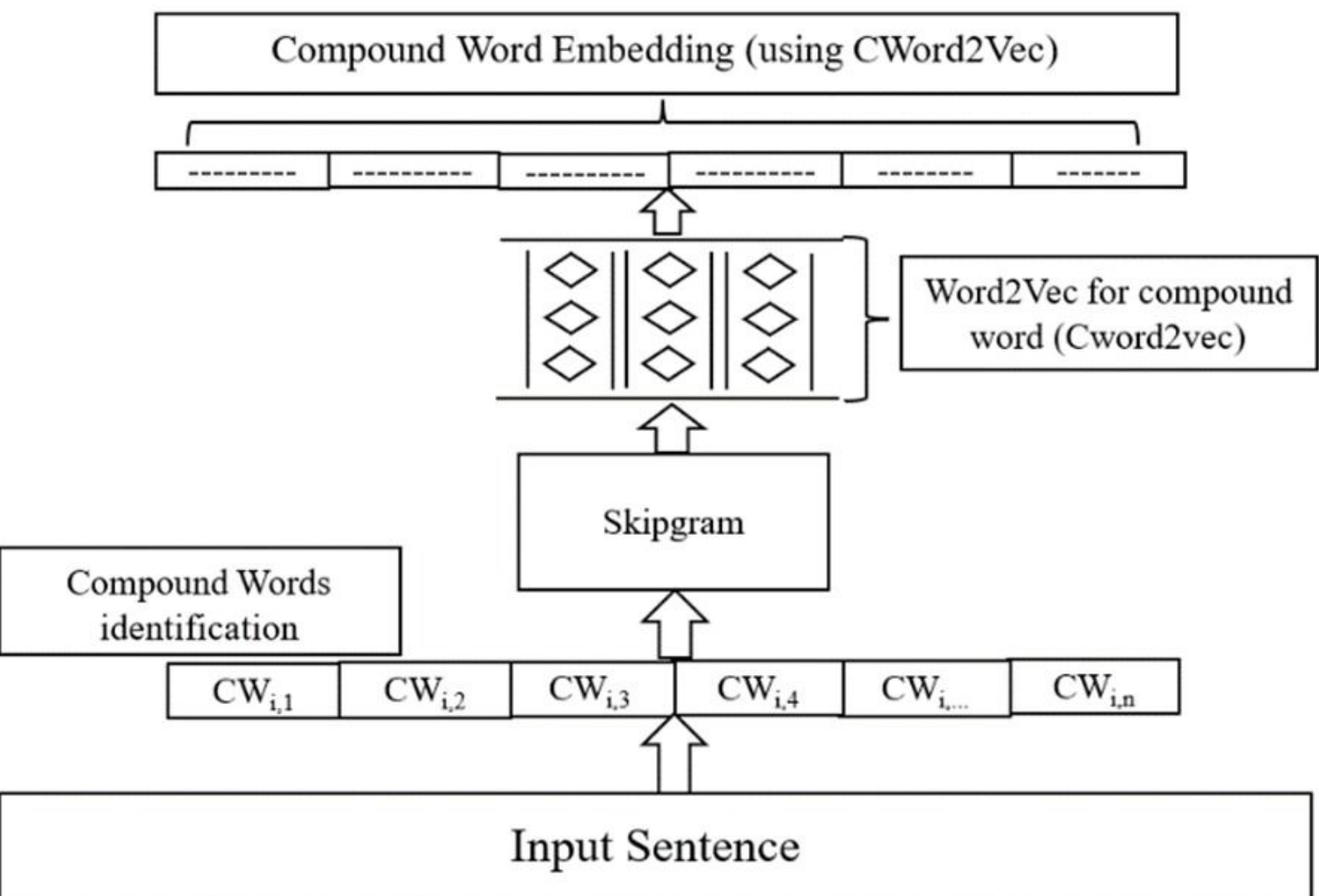

Supplement: Supplemental Information 5 [file peerj-cs-11-2937-s005.pdf]

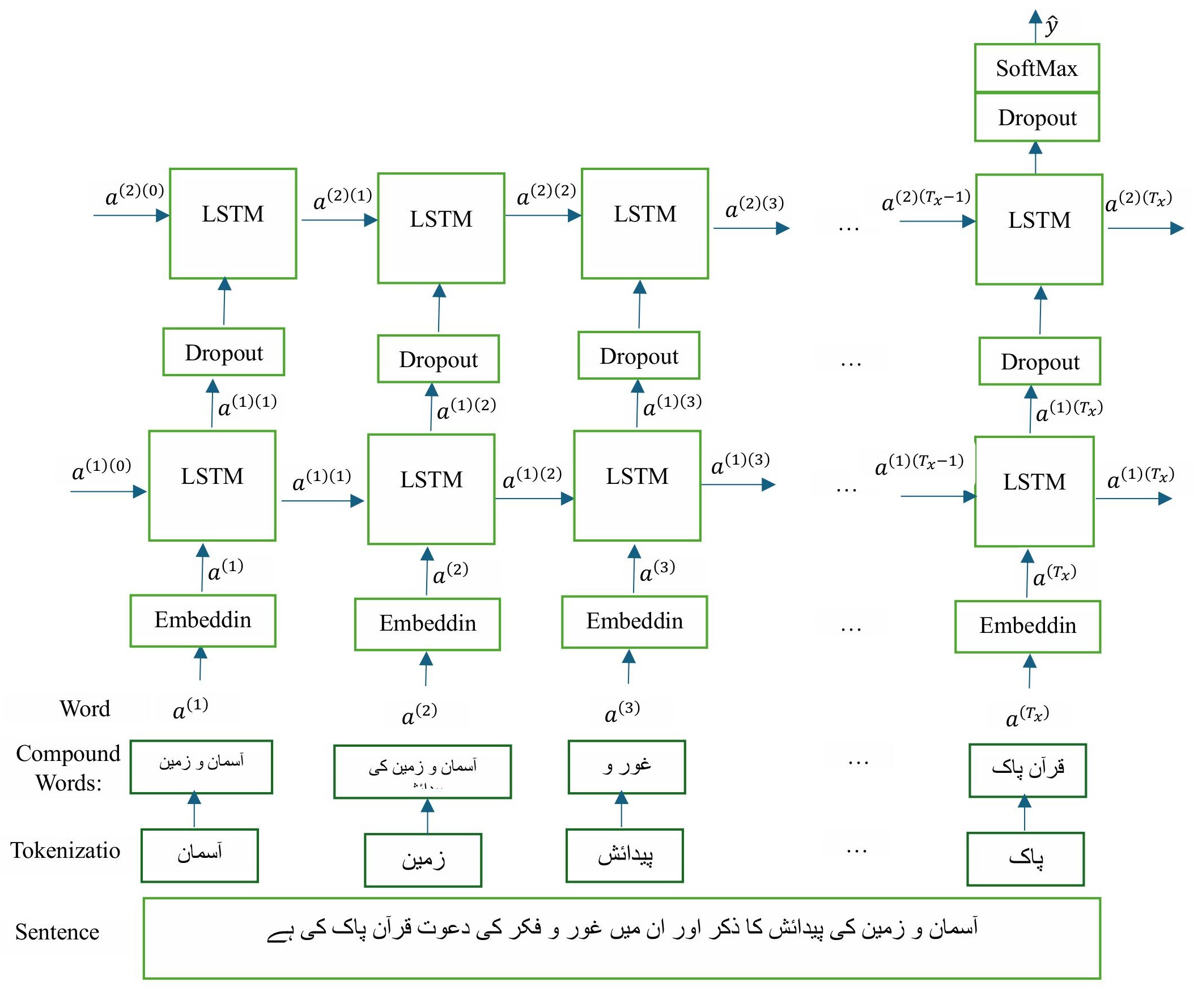

Supplement: Supplemental Information 6 [file peerj-cs-11-2937-s006.jpg]

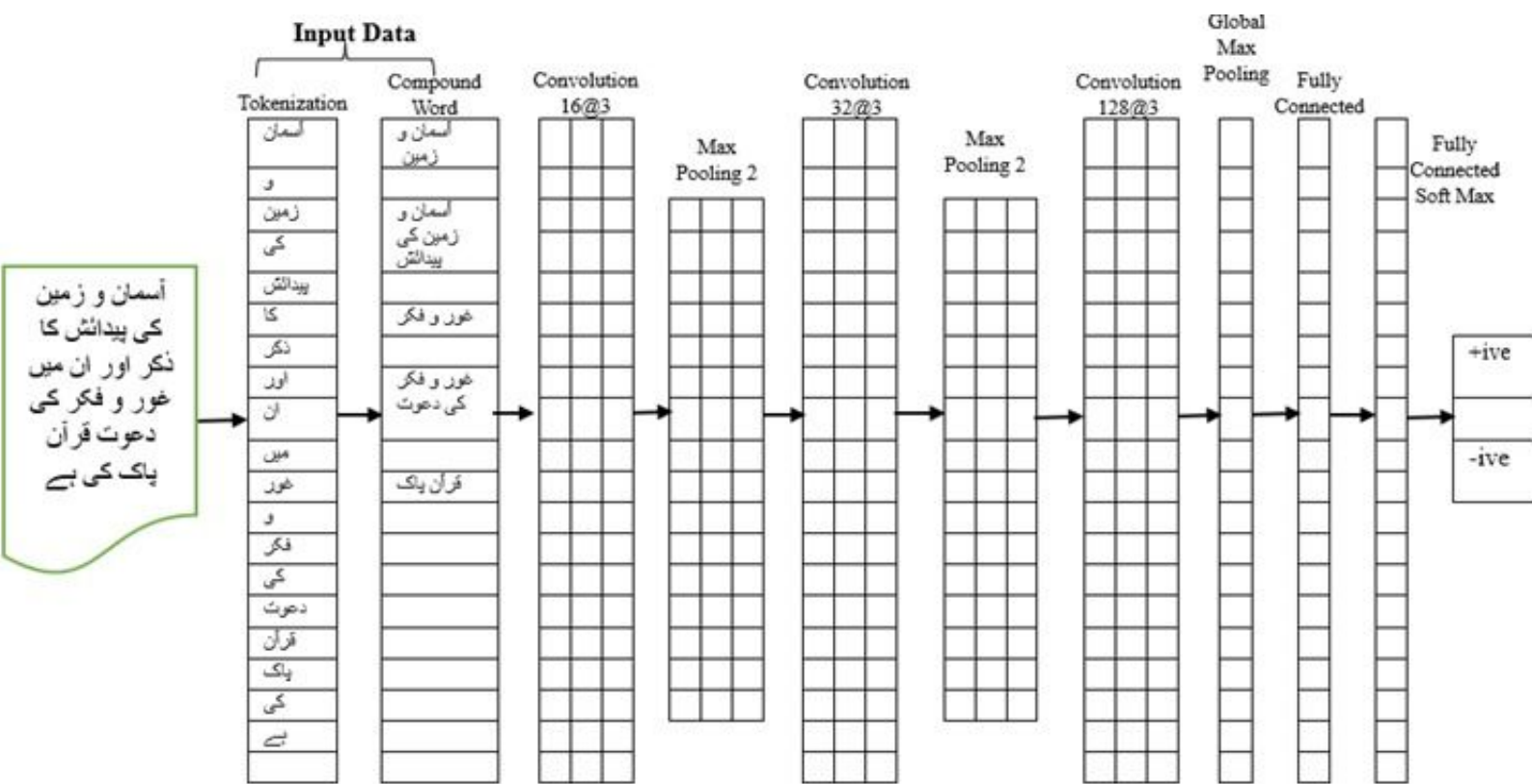

Supplement: Supplemental Information 7 [file peerj-cs-11-2937-s007.pdf]
