# Supplementary material for: Cword2vec: a novel morphological rule-based word embedding approach for Urdu text sentiment analysis
Source: PeerJ Comput Sci. 2025 Jul 15;11:e2937. doi: 10.7717/peerj-cs.2937 (PMC12453651; doi:10.7717/peerj-cs.2937)
Supplement: Supplemental Information 8 [file peerj-cs-11-2937-s008.pdf]

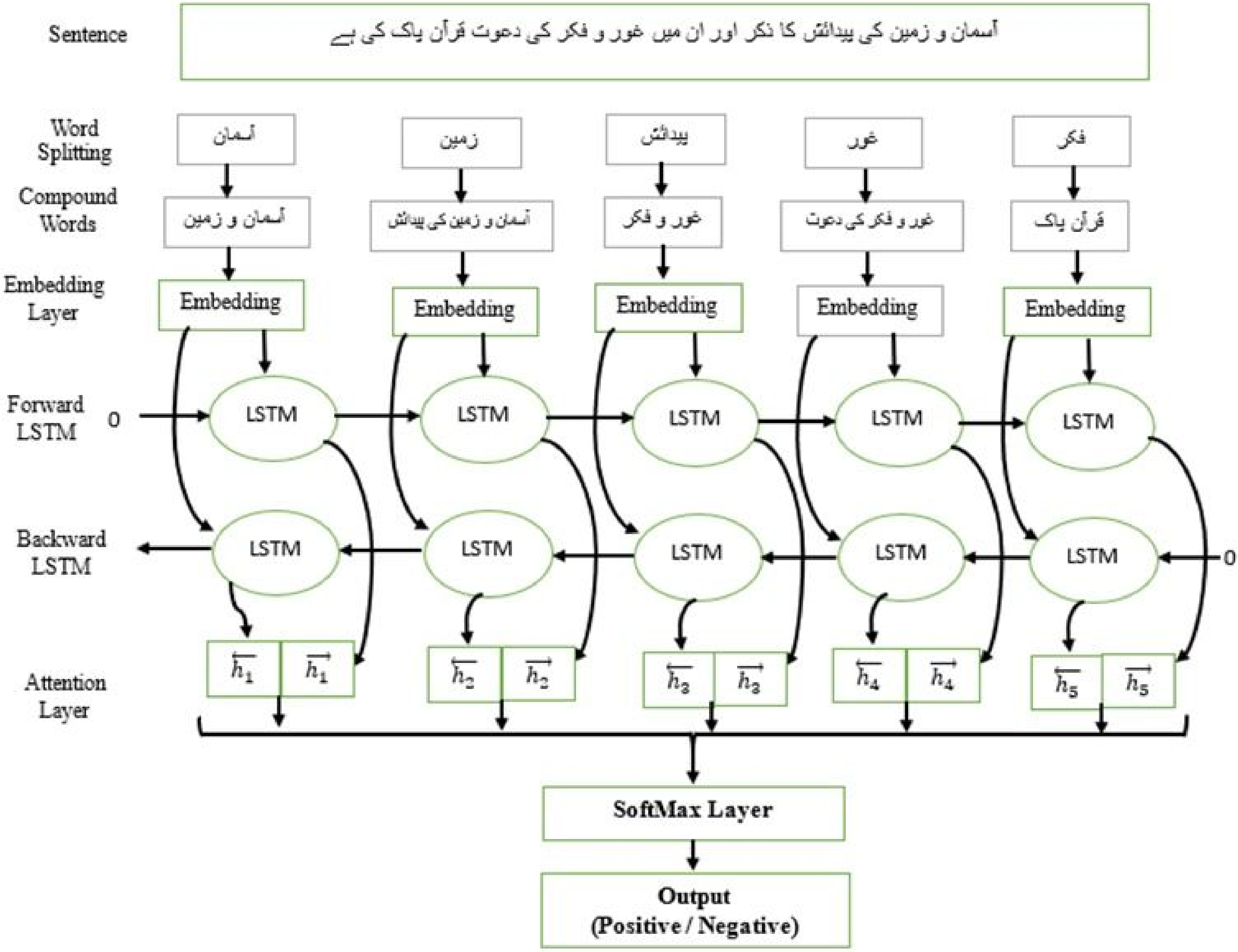

Sentence
آسمان و زمین کی پیدائش کا ذکر اور ان میں غور و فکر کی دعوت قرآن پاک کی ہے
Word Splitting
آسمان
زمین
پیدائش
غور
فکر
Compound Words
آسمان و زمین
آسمان و زمین کی پیدائش
غور و فکر
غور و فکر کی دعوت
قرآن پاک
Embedding Layer
Embedding
Embedding
Embedding
Embedding
Embedding
Forward LSTM
0
LSTM
LSTM
LSTM
LSTM
LSTM
Backward LSTM
LSTM
LSTM
LSTM
LSTM
LSTM
0
Attention Layer
$\overleftarrow{h_1}$ $\overrightarrow{h_1}$
$\overleftarrow{h_2}$ $\overrightarrow{h_2}$
$\overleftarrow{h_3}$ $\overrightarrow{h_3}$
$\overleftarrow{h_4}$ $\overrightarrow{h_4}$
$\overleftarrow{h_5}$ $\overrightarrow{h_5}$
SoftMax Layer
Output
(Positive / Negative)
